# Switching from inotersen to eplontersen in patients with hereditary transthyretin-mediated amyloidosis with polyneuropathy: analysis from NEURO-TTRansform

**DOI:** 10.1007/s00415-024-12616-6

**Published:** 2024-08-13

**Authors:** Isabel Conceição, John L. Berk, Markus Weiler, Pedro A. Kowacs, Noel R. Dasgupta, Sami Khella, Chi-Chao Chao, Shahram Attarian, T. Jesse Kwoh, Shiangtung W. Jung, Jersey Chen, Nicholas J. Viney, Rosie Z. Yu, Morie Gertz, Ahmad Masri, Márcia Waddington Cruz, Teresa Coelho

**Affiliations:** 1 ULS Santa Maria, CAML, Instituto de Medicina Molecular, Faculdade de Medicina da Universidade de Lisboa, Lisbon, Portugal; 2 Boston University School of Medicine, Boston, MA USA; 3 Amyloidosis Center and Department of Neurology, Heidelberg University Hospital, Heidelberg, Germany; 4 Instituto de Neurologia de Curitiba, Curitiba, Paraná Brazil; 5 Indiana University School of Medicine, Indianapolis, IN USA; 6 University of Pennsylvania School of Medicine, Philadelphia, PA USA; 7 National Taiwan University Hospital, Taipei, Taiwan; 8 Neuromuscular Disorders and ALS Department, Centre Hospitalier Universitaire La Timone, Marseille, France; 9 Clinical Development, Ionis Pharmaceuticals, Inc., Carlsbad, CA USA; 10 Late-Stage Development, Cardiovascular, Renal, and Metabolism, BioPharmaceuticals R&D, AstraZeneca, Gaithersburg, MA USA; 11 Preclinical Development, Ionis Pharmaceuticals, Inc, Carlsbad, CA USA; 12 Mayo Clinic, Rochester, MN USA; 13 OHSU Center for Hypertrophic Cardiomyopathy and Amyloidosis, Portland, OR USA; 14 CEPARM, Amyloidosis Center, University Hospital, Federal University of Rio de Janeiro, Rio de Janeiro, Brazil; 15 Centro Hospitalar Universitário de Santo António, Porto, Portugal

**Keywords:** Amyloidosis, Treatment efficacy, Treatment safety, Antisense oligonucleotide, Transthyretin

## Abstract

**Background:**

The phase 3 NEURO-TTRansform trial showed eplontersen treatment for 65 weeks reduced transthyretin (TTR), halted progression of neuropathy impairment, and improved quality of life (QoL) in adult patients with hereditary TTR-mediated amyloidosis with polyneuropathy (ATTRv-PN), vs. historical placebo.

**Methods:**

NEURO-TTRansform enrolled patients with ATTRv-PN. A subset of patients were randomized to receive subcutaneous inotersen 300 mg weekly (Weeks 1–34) and subsequently switched to subcutaneous eplontersen 45 mg every 4 weeks (Weeks 37–81). Change in serum TTR and treatment-emergent adverse events (TEAEs) were evaluated through Week 85. Effects on neuropathy impairment, QoL, and nutritional status were also evaluated.

**Results:**

Of 24 patients randomized to inotersen, 20 (83%) switched to eplontersen at Week 37 and four discontinued due to AEs/investigator decision. Absolute change in serum TTR was greater after switching from inotersen (−74.3%; Week 35) to eplontersen (−80.6%; Week 85). From the end of inotersen treatment, neuropathy impairment and QoL were stable (i.e., did not progress) while on eplontersen, and there was no deterioration in nutritional status. TEAEs were fewer with eplontersen (Weeks 37–85; 19/20 [95%] patients) compared with inotersen (up to Week 35; 24/24 [100%] patients). Mean platelet counts decreased during inotersen treatment (mean nadir reduction ‒40.7%) and returned to baseline during eplontersen treatment (mean nadir reduction, ‒3.2%).

**Conclusions:**

Switching from inotersen to eplontersen further reduced serum TTR, halted disease progression, stabilized QoL, restored platelet count, and improved tolerability, without deterioration in nutritional status. This supports a positive benefit-risk profile for patients with ATTRv-PN who switch from inotersen to eplontersen.

**Supplementary Information:**

The online version contains supplementary material available at 10.1007/s00415-024-12616-6.

## Introduction

Hereditary transthyretin amyloidosis with polyneuropathy (ATTRv-PN) is a rare, progressive, irreversible, and life-threatening autosomal dominant disorder predominantly caused by single-point mutations in the transthyretin (*TTR*) gene encoding TTR, a thyroxine and vitamin A transporter [1–4]. In ATTRv-PN, abnormal TTR proteins misfold and aggregate into extracellular amyloid deposits in peripheral and autonomic nerves and other major organs [4], leading to progressive polyneuropathic impairment, deterioration in quality of life (QoL), and poor prognosis. Death from complications of amyloid cardiomyopathy or cachexia typically occur within 12 years of symptom onset [2, 5–9].

In addition to liver transplantation, other therapeutic approaches have included tafamidis and diflunisal, which stabilize the native TTR tetrameric structure and slow disease progression [10, 11]. In addition, four gene-silencing treatments (patisiran, inotersen, vutrisiran, and most recently, eplontersen) are available. Patisiran requires intravenous infusion every 3 weeks and premedication with intravenous (IV) corticosteroid (e.g., dexamethasone or equivalent), oral acetaminophen, IV H1 blocker (e.g., diphenhydramine 50 mg or equivalent) and IV H2 blocker (e.g., famotidine 50 mg or equivalent) [12], while inotersen requires once-weekly (QW; 300 mg subcutaneous [SC]) dosing, and regular laboratory assessment for thrombocytopenia and glomerulonephritis [13]. Vutrisiran benefits from less frequent dosing than patisiran or inotersen but, like all *TTR* silencers, monitoring for ocular symptoms suggestive of vitamin A deficiency and use of vitamin A supplementation is advised [14]. More efficacious disease-modifying agents are needed providing more potent *TTR* silencing, greater convenience of use, and more favorable tolerability [15].

Eplontersen—an antisense oligonucleotide (ASO) recently approved in the United States for use in adults with ATTRv-PN—is a triantennary N-acetyl galactosamine (GalNAc)-conjugated ASO designed for receptor-mediated uptake by hepatocytes [16]. Significantly, GalNAc conjugation was shown to increase the potency of ASO molecules by 30- and 50-fold in mice expressing a mutated human genomic *TTR* sequence and in human hepatocyte cell culture, respectively [16], primarily due to increased uptake by hepatocytes [17]. Thus, eplontersen is used at a comparatively lower dose than the unconjugated inotersen (45 mg SC, every 4 weeks [Q4W] vs. 284 mg [free acid equivalent] SC, QW; ~ 25-fold) [18]. This provides potential for improved tolerability [17]. The phase 3 open-label NEURO-TTRansform trial (NCT04136184) met its primary endpoints, demonstrating that eplontersen treatment reduced serum TTR, halted progression of neuropathy impairment, and improved QoL in patients with ATTRv-PN, compared with historical placebo from the NEURO-TTR trial (NCT01737398) of inotersen [19, 20]. NEURO-TTRansform’s design included a randomized inotersen treatment reference group, which allowed cross-trial comparison of disease progression between NEURO-TTRansform and NEURO-TTR, supporting the use of the historical placebo [15, 19].

In this further analysis from NEURO-TTRansform, efficacy and safety in the subgroup of patients randomized to receive inotersen and who subsequently switched to eplontersen was investigated.

## Methods

### Trial design

The design, methodology, and characteristics of participants in the NEURO-TTRansform trial (NCT04136184/EudraCT 2019-001698-10) have been reported previously [15, 21]. The trial design is shown in Supplementary Fig. 1. Briefly, patients were randomized 1:6 to open-label inotersen 300 mg SC QW (Weeks 1–34) followed by a switch to eplontersen 45 mg SC Q4W (Weeks 37–81) or continuous eplontersen 45 mg SC Q4W (Weeks 1–81).

The relevant institutional review boards or independent ethics committees approved the trial protocol and amendments. NEURO-TTRansform was conducted in accordance with the International Council for Harmonisation and Good Clinical Practice guidelines, and relevant country-specific laws. All patients provided written informed consent before enrollment.

### Patients

Eligible patients were aged 18–82 years, with ATTRv-PN Stage 1 or 2 according to the familial amyloid polyneuropathy or Coutinho stage, a documented genetic mutation in the *TTR* gene, and symptoms and signs consistent with neuropathy associated with TTR-mediated amyloidosis, including a Neuropathy Impairment Score of ≥ 10 and ≤ 130. Patients had to be willing to adhere to daily vitamin A supplementation, per protocol. Detailed inclusion and exclusion criteria have been reported previously [15]. The same eligibility criteria were used in the NEURO-TTR trial.

### Assessments

#### Efficacy

##### Serum TTR

Mean percentage change from baseline in serum TTR concentration was evaluated at predefined intervals during the treatment period, at Weeks 5, 9, 13, 25, and 35 (inotersen treatment period), and at Weeks 49, 57, 65, 73, 81, and 85 (eplontersen treatment period). Analysis was performed using a novel validated electrochemiluminescence (ECL) assay.

##### Disease progression and QoL

Disease progression and QoL were assessed using validated instruments as change from baseline in the modified Neuropathy Impairment Score + 7 (mNIS + 7) composite score, and Norfolk Quality of Life-Diabetic Neuropathy (Norfolk QoL-DN) total score at Week 35 (inotersen treatment period), and at Weeks 66 and 85 (eplontersen treatment period). mNIS + 7 composite score ranges from − 22.3 to 346.3, with higher scores indicating poorer function; a decrease in score indicates improvement [22]. Norfolk QoL-DN total score ranges from − 4 to 136, with a higher score indicative of poorer QoL; a decrease in score indicates improvement [20].

##### Nutritional status

Nutritional status was assessed as change from baseline in modified body mass index (mBMI; BMI [kg/m^2^] × serum albumin [g/L]), where higher values are indicative of better nutritional status. mBMI was assessed at baseline, at Weeks 13 and 35 (inotersen treatment period), and Weeks 66 and 85 (eplontersen treatment period). mBMI was utilized to avoid potential discrepancies that can be seen with BMI measurement due to a low serum albumin level and fluid retention [23].

#### Safety

Safety assessments were evaluated throughout the study, as described previously [15, 19]. This included monitoring of treatment-emergent adverse events (TEAEs). TEAEs were further classified as being of special interest (ocular adverse events [AEs] related to vitamin A deficiency, thrombocytopenia, and glomerulonephritis) or as other AEs of interest (including injection-site reactions, flu-like symptoms, and abnormal liver function).

Clinical laboratory safety (including chemistry, hematology, urinalysis, thyroid panel, inflammatory panel, and coagulation) was evaluated along with vital signs and weight, physical examinations, electrocardiograms, and an ocular questionnaire.

#### Pharmacokinetics and immunogenicity

Plasma inotersen concentrations were measured at Weeks 1 through 35 using a validated quantitative hybridization-based enzyme-linked immunosorbent assay (ELISA) method (lower limit of quantification [LLOQ] 1 ng/mL). Plasma eplontersen concentrations, measured at Week 49 and thereafter, were determined using a validated and quantitative hybridization-based assay with ECL detection (hybridization ECL) assay (LLOQ 0.129 ng/mL). Concentrations measured during the eplontersen treatment period reflected total oligonucleotide concentration. Analyses were performed by PPD Bioanalytical Laboratory, Richmond, VA, USA.

Non-compartmental pharmacokinetic analysis of inotersen and eplontersen concentrations was conducted. Limited intensive plasma pharmacokinetic samples were collected following dosing on Days 1, 225, and 449. Plasma trough concentration (C_trough_) and post-treatment plasma concentrations of inotersen and eplontersen were evaluated in all patients, while area under the concentration–time curve (AUC_0–6h_), maximum plasma concentration (C_max_), and time to C_max_ (T_max_) were evaluated in a subset of 35 patients (28 who were randomized to eplontersen and seven who were randomized to inotersen); C_max_ and T_max_ were obtained directly from concentration–time data.

Plasma samples collected throughout the study (predose [baseline, Day 1], and on Days 29, 85, 225, 337, 449, 589, and 729, including early termination samples) were analyzed for inotersen and eplontersen antidrug antibodies (ADAs) using validated qualitative ELISA methods (QPS Holdings LLC, Newark, USA [inotersen] and Charles River Laboratories, Quebec, Canada [eplontersen]) in a multitiered approach, which consisted of screening, and confirmatory and titer assays. Plasma samples collected at baseline (predose on Day 1) were analyzed for both anti-inotersen and anti-eplontersen antibodies. During the inotersen treatment period (Weeks 1‒34), plasma samples were analyzed for anti-inotersen antibodies. Following the switch to eplontersen treatment (Week 37 onwards), samples were analyzed for both anti-inotersen and anti-eplontersen antibodies.

### Statistical analysis

All outcomes are summarized descriptively by treatment received before and after treatment switch. Immunogenicity status was summarized using descriptive statistics, and range by treatment and dose, and ADA levels as peak titer quartiles.

## Results

### Patients

Of the 24 patients randomized to inotersen, four patients discontinued study drug due to AEs (*n* = 3) or investigator decision (*n* = 1); 20 (83.3%) patients switched from inotersen to eplontersen at Week 37; there were no notable differences in baseline demographics and clinical characteristics following exclusion of these four patients from the analysis (Table 1).

**Table 1:** Baseline demographics and disease characteristics

	Randomized to inotersen (*n* = 24)	Randomized to inotersen and switched to eplontersen (*n* = 20)	Randomized to eplontersen (*n* = 144)	Historical placebo (*n* = 60)
Demographics				
Mean age, years (SD)	51.1 (14.4)	51.4 (14.8)	53.0 (15.0)	59.5 (14.1)
Age group, *n* (%)	24 (100)	20 (100)	144 (100)	60 (100)
< 65 years	16 (66.7)	13 (65.0)	100 (69.4)	34 (56.7)
65–74 years	7 (29.2)	6 (30.0)	36 (25.0)	17 (28.3)
Male, *n* (%)	16 (66.7)	13 (65.0)	100 (69.4)	41 (68.3)
Race, *n* (%)	23 (95.8)	20 (100)	143 (99.3)	60 (100)
Asian	2 (8.7)	2 (10.0)	22 (15.4)	3 (5.0)
Black or African American	0	0	5 (3.5)	1 (1.7)
White	19 (82.6)	16 (80.0)	112 (78.3)	53 (88.3)
Other or multiple	2 (8.7)	2 (10.0)	4 (2.8)	3 (5.0)
Region, *n* (%)	24 (100)	20 (100)	144 (100)	60 (100)
Europe	10 (41.7)	7 (35.0)	54 (37.5)	23 (38.3)
North America	5 (20.8)	5 (25.0)	21 (14.6)	26 (43.3)
South America/Australasia/Asia	9 (37.5)	8 (40.0)	69 (47.9)	11 (18.3)
Clinical characteristics				
Duration of disease from diagnosis of ATTRv-PN, years, median (range)	2.4 (0.2, 19.3)	2.2 (0.2, 19.3)	2.5 (0.0, 31.6)	2.0 (0.1, 13.3)
Duration of disease from onset of symptoms of ATTRv-PN, years, median (range)	3.5 (0.7, 47.3)	3.3 (0.7, 47.3)	4.5 (0.4, 29.5)	4.0 (0.7, 23.1)
Coutinho stage, *n* (%)	24 (100)	20 (100)	144 (100)	60 (100)
1 (ambulatory)	18 (75.0)	15 (75.0)	115 (79.9)	42 (70.0)
2 (requires ambulatory support)	6 (25.0)	5 (25.0)	29 (20.1)	18 (30.0)
Positive ATTRv-CM diagnosis	7 (29.2)		39 (27.1)	22 (36.7)
mNIS + 7 composite score, mean (SE)	65.1 (6.8)	65.6 (8.2)	81.3 (3.6)	74.8 (5.0)
Norfolk QoL-DN total score, mean (SE)	40.1 (4.4)	37.3 (4.9)	44.1 (2.3)	48.7 (3.5)
BMI, kg/m^2^, mean (SD)	26.4 (5.4)	26.2 (4.7)	24.4 (4.9)	24.2 (4.9)
mBMI^a^ kg/m^2^ × albumin g/L, mean (SD)	1101.7 (246.5)	1098.8 (236.7)	1025.8 (235.1)	1049.9 (228.4)
V30M *TTR* variant, *n* (%)	16 (66.7)	12 (60.0)	85 (59.0)	33 (55.0)
Previous treatment with tafamidis or diflunisal	15 (62.5)	11 (55.0)	100 (69.4)	36 (60.0)

*ATTRv-CM* ATTRv cardiomyopathy, *ATTRv-PN* ATTRv polyneuropathy, *BMI* body mass index, *mBMI* modified BMI, *mNIS + 7* modified Neuropathy Impairment Score + 7, *Norfolk QoL-DN* Norfolk Quality of Life-Diabetic Neuropathy, *SD* standard deviation, *SE* standard error, *TTR* transthyretin

^a^Defined as BMI (kg/m^2^) × albumin level (g/L); higher scores are indicative of better nutritional status

In those patients initially randomized to inotersen (n = 24), most were male (66.7%) and White (82.6%), with V30M *TTR* variant (66.7%); 62.5% had received prior treatment with tafamidis or diflunisal. For patients randomized to inotersen, mean (standard deviation [SD]) age was 51.1 (14.4) years, BMI was 26.4 (5.4) kg/m^2^, mBMI was 1101.7 (246.5) kg/m^2^ × albumin level (g/L), and median duration of disease from onset of symptoms was 3.5 (range 0.7–47.3) years. At baseline, mNIS + 7 and Norfolk QoL-DN scores were lower for the inotersen randomized group relative to the eplontersen randomized or historical placebo groups (Table 1).

All patients received concomitant vitamin A supplementation, as per the study protocol.

### Outcomes

#### Efficacy

##### Serum TTR

Following initiation of treatment with inotersen, mean serum TTR levels were reduced from baseline by − 52.3%, − 79.7%, − 78.8%, and − 74.3% at Weeks 5, 13, 25, and 35, respectively. Following switch to eplontersen at Week 37, mean serum TTR levels were further reduced, with mean percentage reductions from baseline of − 79.9% and − 80.6% at Weeks 65 and 85, respectively.

##### Disease progression and QoL

**Fig. 1:**
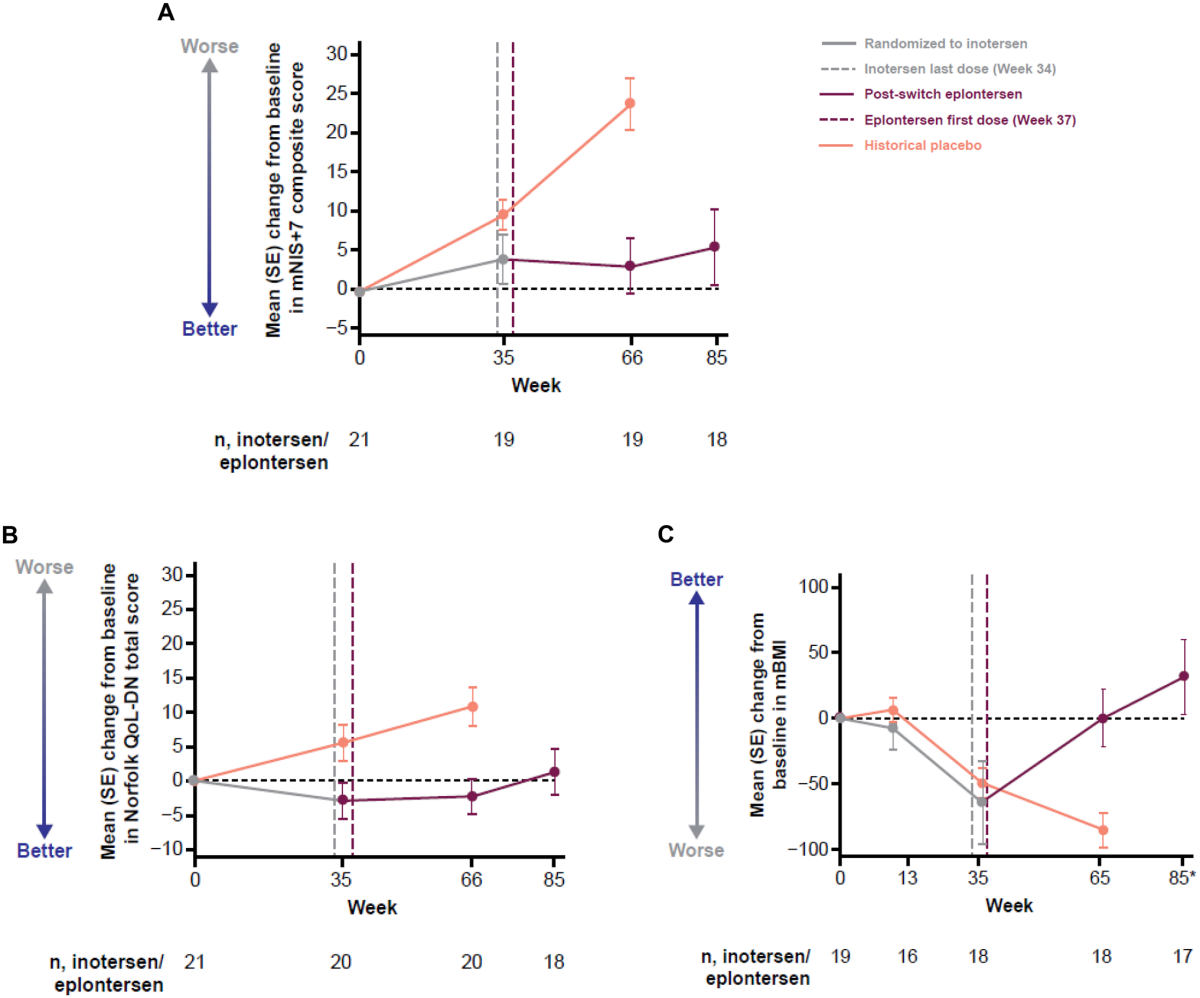
Change from baseline in **A** mNIS + 7 composite score, **B** Norfolk QoL-DN total score, and **C** mBMI over time with inotersen and after switching to eplontersen. mNIS + 7 composite scores range from − 22.3 to 346.3; higher scores indicate poorer function, while a decrease in score indicates improvement. *Week 85 is based on nominal visit; includes all data collected at the Week 85 visit without a visit window. Norfolk QoL-DN total scores range from − 4 to 136; higher scores are indicative of poorer QoL, while a decrease in score indicates improvement. mBMI increase is indicative of improved nutritional status, while a decrease denotes worsening. Historical placebo is from the NEURO-TTR study [20]. *mBMI* modified body mass index, *mNIS + 7* modified Neuropathy Impairment Score + 7, *Norfolk QoL-DN* Norfolk Quality of Life-Diabetic Neuropathy, *QoL* quality of life, *SE* standard error

Assessment of neuropathy impairment based on mNIS + 7 composite scores is shown in Fig. 1A. Disease progression appeared slowed during treatment with inotersen (baseline to Week 35) and was halted during eplontersen treatment (Weeks 37‒85), in contrast to the historical placebo group, which showed progressive decline. Measurement of patient QoL (Norfolk QoL-DN total score) showed stabilization with inotersen that was maintained with eplontersen treatment after switching (Fig. 1B).

#### Nutritional status

During the inotersen treatment period, nutritional status (mBMI) markedly declined from baseline with the same trend and magnitude as the historical placebo group. Marked improvement in mBMI was subsequently seen with eplontersen treatment, post switching with return to baseline (Fig. 1C).

#### Safety

##### AEs

The incidence of TEAEs before and after switching was similar despite differences in the inotersen and eplontersen treatment periods; events were reported in all 24 patients (100%) randomized to inotersen (Weeks 1‒37), and in 19 of 20 patients (95%) after switching to eplontersen treatment (Weeks 37‒85 + ; onset after the first eplontersen dose to discontinuation from post-Week 85 follow-up regardless of inclusion in long-term extension study, or if completed 20-week follow-up) (Table 2). The incidence of serious TEAEs was also similar between the pre-switch inotersen (three patients; 12.5%) and post-switch eplontersen (three patients; 15.0%) treatment periods.

**Table 2:** Summary of TEAEs

Patients, *n* (%)	Randomized to inotersen [Weeks 1–37] (*n* = 24)	Post-switch eplontersen [Weeks 37–85 +] (*n* = 20)
Any TEAE	24 (100)	19 (95)
Related to study drug	17 (71)	6 (30)
Leading to study drug discontinuation	3 (13)	0
Leading to study withdrawal	2 (8)	0
Maximum severity of TEAEs^a^		
Mild	8 (33)	8 (40)
Moderate	13 (54)	7 (35)
Severe	3 (13)	4 (20)
Any serious TEAE	3 (13)	3 (15)
Leading to study drug discontinuation	1 (4)	0
TEAEs of special interest	8 (33)	3 (15)
Ocular events related to vitamin A deficiency	4 (17)	3 (15)
Thrombocytopenia	6 (25)	0
Glomerulonephritis	0	0
Other AEs of interest		
Injection site reactions^b^	12 (50)	0
Flu-like symptoms^c^	4 (17)	0
Abnormal liver function^d^	4 (17)	1 (5)

*AE* adverse event, *TEAE* treatment-emergent adverse event

^a^For severity, patients reporting more than one TEAE are counted only once using the worst severity reported

^b^Defined as TEAEs, with preferred terms containing the text “injection site”

^c^Defined as TEAEs, with the preferred terms “influenza-like illness” or “pyrexia” (or feeling hot or body temperature increased), plus at least one of chills, myalgia, arthralgia, malaise, fatigue, headache, nausea

^d^TEAE within the Standardized MedDRA (Medical Dictionary of Regulatory Activities) Query: drug-related hepatic disorders–comprehensive search

‘ + ’ Includes all AEs with onset after first dose through to when patients discontinued from follow-up; regardless of continuation in any long-term extension study, or where patients did not complete the full follow-up period

Patient distribution by TEAE maximum severity was broadly similar during the inotersen or eplontersen treatment periods, and comparable with the other treatment groups. TEAEs of special interest (those leading to discontinuation/withdrawal from the study) were reported in eight (33%) patients during the inotersen treatment period and in three (15%) patients during the eplontersen treatment period. Notably, there were no cases of thrombocytopenia reported during the eplontersen treatment period. There were no cases of glomerulonephritis reported in patients randomized to inotersen or after switching to eplontersen. Ocular events related to vitamin A deficiency were reported in four (17%) patients during the inotersen treatment period and in three (15%) patients during the eplontersen treatment period (Table 2). During the eplontersen treatment period, there were no reports of injection-site reaction or flu-like symptoms reported, and one reported event of elevated transaminases.

Three (13%) patients discontinued inotersen treatment early due to a TEAE; none discontinued following switch to eplontersen. Similarly, one (4%) patient discontinued inotersen treatment due to a serious TEAE (*n* = 3: hyperthyroidism, drug eruption, nephroangiosclerosis; *n* = 1: investigator judgment, persistent tubulointerstitial nephritis, and proteinuria) with none in the post-switch eplontersen group. One patient who switched to eplontersen discontinued treatment early (voluntary withdrawal; reason not reported).

##### Laboratory safety measures

**Fig. 2:**
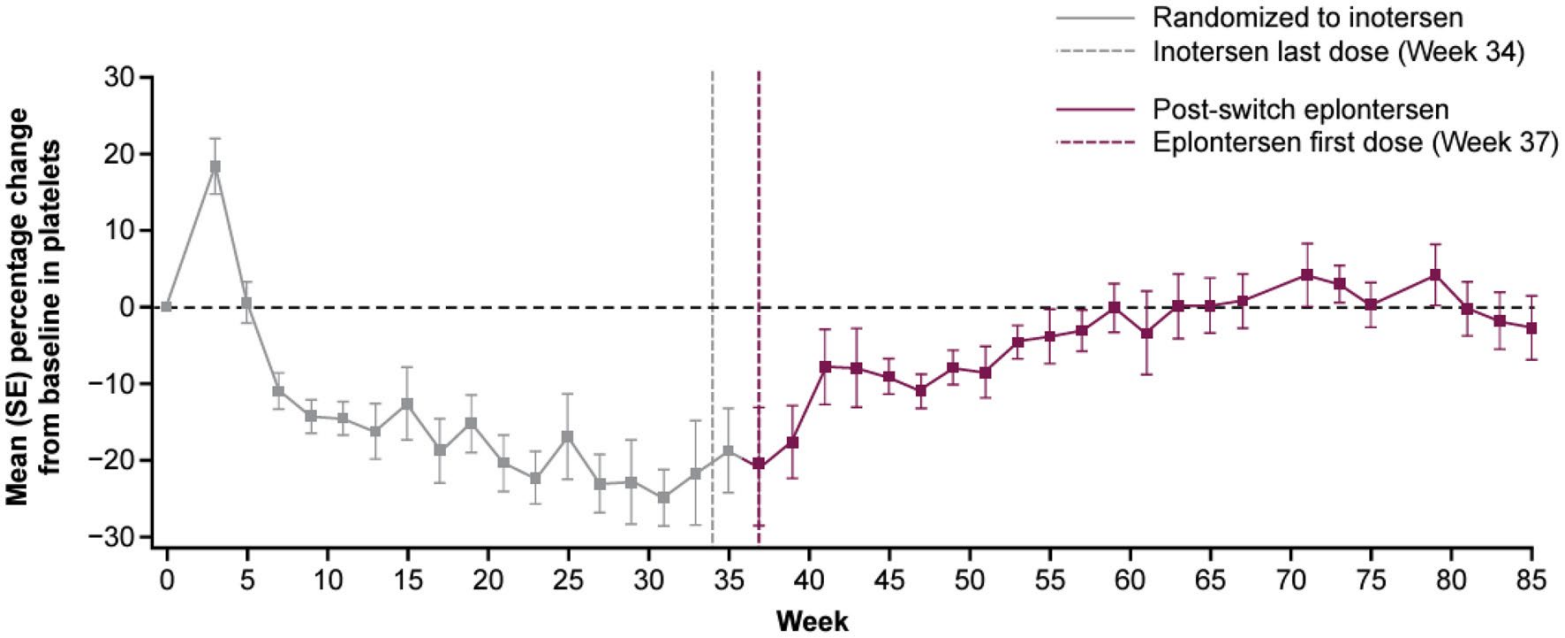
Percentage change in platelet count with inotersen and after switching to eplontersen. Inotersen: 300 mg once weekly (Weeks 1−34); eplontersen: 45 mg once every 4 weeks (Weeks 37−81). *SE* standard error

At baseline, mean (SD) platelet count was 228.6 (67.0) × 10^9^/L. During the inotersen treatment period, a gradual reduction in platelet count was seen, with a mean (SD) reduction in platelet count at Week 35 from baseline of – 18.8 (22.8)% (– 44.5 [58.8] 10^9^/L) (Fig. 2). Mean nadir reduction was ‒ 40.7%. After switching to eplontersen, mean platelet count increased over a 22-week period to baseline level and remained stable thereafter (mean nadir reduction, ‒ 3.2%). The incidence of patients with a platelet count below the lower limit of normal (< 140 × 10^9^/L) at any time post-baseline was 54.2% (13/24 patients) during the inotersen treatment period and 35.0% (7/20 patients) during the eplontersen treatment period. No patient, regardless of treatment received, had a platelet count < 25 × 10^9^/L and no patient discontinued treatment or was withdrawn from the study due to low platelet count.

In patients randomized to inotersen, there was an observed reduction in mean estimated glomerular filtration rate (eGFR), reaching a nadir of ~ 10 mL/min/1.73 m^2^ below baseline; following the switch to eplontersen, mean eGFR remained stable at the level at switch; ~ 5 mL/min/1.73 m^2^ below [inotersen] baseline (Supplementary Fig. 2).

#### Pharmacokinetics and immunogenicity

On Day 225 of the inotersen treatment period, C_max_ and AUC_0–6 h_ (exposure to study drug) was 8.47 μg/mL and 46.1 μg*h/mL, respectively. On Day 449 of the eplontersen treatment period, these values were 0.27 μg/mL and 1.21 μg*h/mL, respectively; mirroring the 6.3-fold lower dose and 4-fold lower frequency of dosing of eplontersen (Table 3). At the same timepoints, plasma C_trough_ level was 55.6 ng/ml for inotersen and 1.35 ng/ml for eplontersen, while median T_max_ was 3.5 h (range: 3.0–4.0 h) and 3.1 h (range: 2.1–6.0 h), respectively.

**Table 3:** Plasma PK parameters of eplontersen and inotersen

Day (treatment, dose)	PK parameter	*n*	Statistic	Value
Randomized to inotersen Inotersen [Weeks 1–37]; eplontersen [Weeks 37–85 +]				
1 Inotersen, 284 mg SC	C_max_ (μg/mL)	6	Geometric mean (SD) Geometric %CV	6.61 (1.87) 69.2
	T_max_ (h)	6	Median Min, max	3.50 2.00, 4.00
	AUC_0–6 h_ (μg*h/mL)	6	Geometric mean (SD) Geometric %CV	28.6 (1.80) 63.9
225 Inotersen, 284 mg SC	C_max_ (μg/mL)	2	Geometric mean (SD) Geometric %CV	8.47 (NA) NA
	T_max_ (h)	2	Median Min, max	3.50 3.00, 4.00
	AUC_0–6 h_ (μg*h/mL)	1	Geometric mean (SD) Geometric %CV	46.1 (NA) NA
	C_trough_ (ng/mL)	12	Median (IQR)	55.6 (44.8, 226)
449 Eplontersen, 45 mg SC	C_max_ (μg/mL)	6	Geometric mean (SD) Geometric %CV	0.27 (2.45) 111
	T_max_ (h)	6	Median Min, max	3.05 2.13, 5.95
	AUC_0–6 h_ (μg*h/mL)	6	Geometric mean (SD) Geometric %CV	1.21 (2.45) 111
	C_trough_ (ng/mL)	19	Median (IQR)	1.35 (0.72, 3.68)
Randomized to eplontersen [Weeks 1–85 +]				
1 Eplontersen, 45 mg SC	C_max_ (μg/mL)	23	Geometric mean (SD) Geometric %CV	0.20 (2.42) 109
	T_max_ (h)	23	Median Min, max	2.17 1.00, 6.00
	AUC_0–6 h_ (μg*h/mL)	23	Geometric mean (SD) Geometric %CV	0.87 (2.28) 98.9
225 Eplontersen, 45 mg SC	C_max_ (μg/mL)	19	Geometric mean (SD) Geometric %CV	0.22 (2.06) 83.0
	T_max_ (h)	19	Median Min, max	2.05 0.97, 6.00
	AUC_0–6 h_ (μg*h/mL)	18	Geometric mean (SD) Geometric %CV	0.84 (1.91) 72.1
	C_trough_ (ng/mL)	123	Median (IQR)	0.20 (0.14, 0.28)
449 Eplontersen, 45 mg SC	C_max_ (μg/mL)	25	Geometric mean (SD) Geometric %CV	0.20 (2.13) 87.7
	T_max_ (h)	25	Median Min, max	2.00 1.00, 6.00
	AUC_0–6 h_ (μg*h/mL)	25	Geometric mean (SD) Geometric %CV	0.83 (1.92) 72.8
	C_trough_ (ng/mL)	121	Median (IQR)	0.27 (0.17, 0.46)

*%CV* percent coefficient of variation, *AUC*_*0–6 h*_ area under the concentration–time curve from 0 to 6 h, *C*_*max*_ maximum plasma concentration, *C*_*trough*_ predose plasma concentration, *h* hours, *IQR* interquartile range, *max* maximum, *min* minimum, *NA* not applicable, *PK* pharmacokinetic(s), *SC* subcutaneous, *SD* standard deviation, *T*_*max*_ time to maximum concentration in plasma

‘ + ’ Includes all adverse events with onset after first dose through to when patients discontinued from follow-up; regardless of continuation in any long-term extension study, or where patients did not complete the full follow-up period

**Fig. 3:**
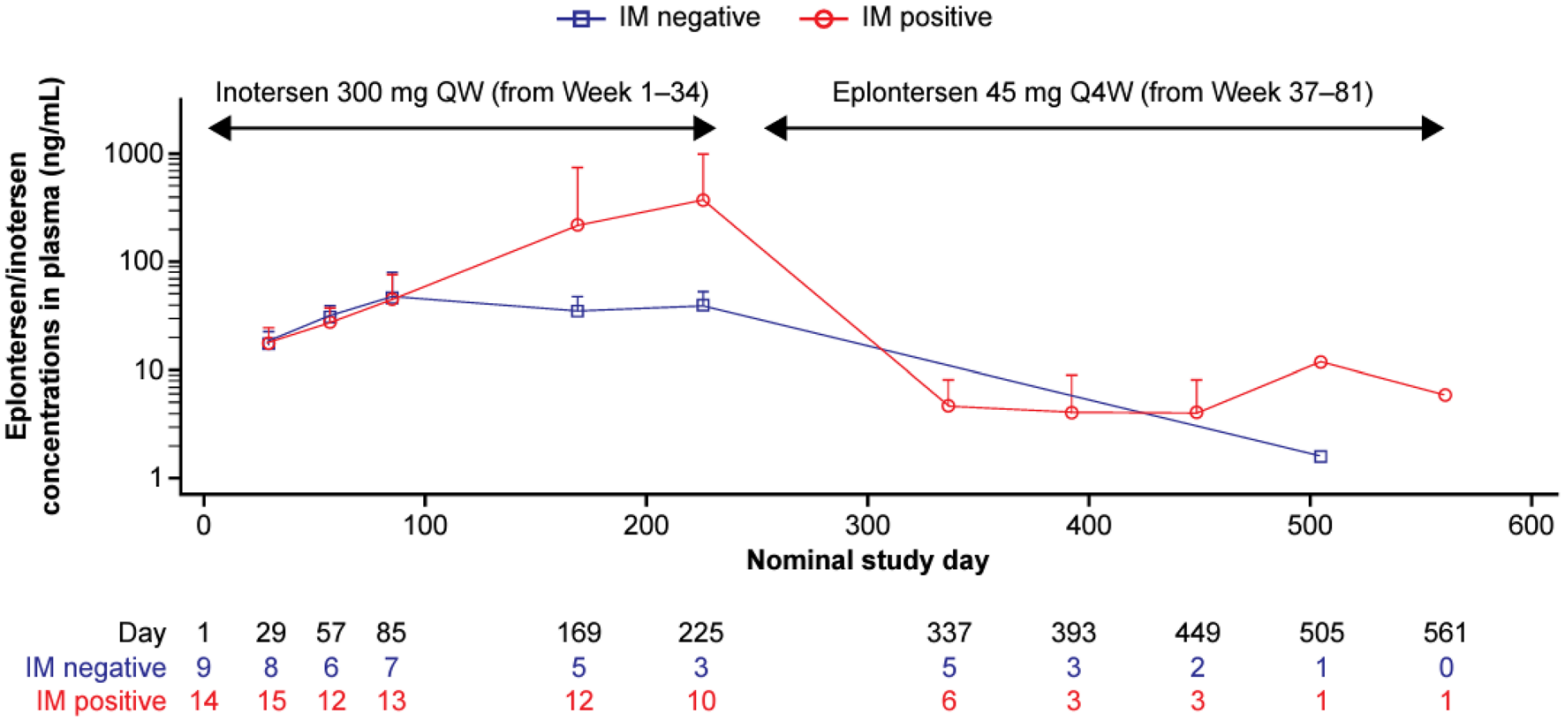
Plasma trough concentration of inotersen and eplontersen over time by IM status (pharmacokinetic set). Values are presented as mean (SD). If plasma concentration was < LLOQ (0.129 ng/mL eplontersen; 1 ng/mL inotersen), value was deemed to be 0; any summary mean < LLOQ was excluded. Samples were excluded for large deviations between scheduled and actual sampling times (difference > 30%) or for large deviations between actual dose and nominal dose (difference > 30%). IM status was defined as positive if any sample from a patient was positive to anti-eplontersen or anti-inotersen antibodies at any time during the treatment or post-treatment evaluation periods (the converse is true for negative IM). *IM* immunogenicity, *LLOQ* lower limit of quantitation, *Q4W* once every 4 weeks, *QW* once weekly, *SD* standard deviation

Inotersen ADAs were detected in 66.7% (16/24) of patients, with treatment-emergent ADAs detected in 62.5% (15/24) of patients (nearly all persistent), with levels remaining stable through to Week 85. Eplontersen ADAs were detected in 58.3% (14/24) of patients, with treatment-emergent ADAs in 54.2% (13/24) of patients. During the inotersen treatment period, time to steady-state (from plasma C_trough_ in immunogenic-negative patients) appeared to be reached by Day 85 (Fig. 3), while during the eplontersen treatment period, C_trough_ was decreased by nearly two orders of magnitude; collectively mirroring change in dosage and frequency. Notably, eplontersen plasma concentration–time profiles in the first 6 h after dose administration were similar between ADA-negative and ADA-positive patients on all examined days (Days 1, 225, and 449), indicating that ADA had minimal effect on peak exposure (data not shown).

## Discussion

This prespecified analysis of the phase 3 NEURO-TTRansform trial demonstrates over 85 weeks a clear positive benefit-risk profile in the subset of patients with ATTRv-PN who were switched from inotersen to eplontersen. Following the switch from inotersen to eplontersen, there was further reduction in mean serum TTR levels (− 80.6%) at Week 85, which was consistent with the reduction seen for eplontersen treatment in the NEURO-TTRansform trial at Week 85 (− 81.8%) [19], and apparent benefit towards neuropathy impairment, QoL, nutritional status, and overall safety and tolerability, including restoration of platelet count to baseline levels.

Significantly, relative to historical placebo which showed a progressive decline, disease progression measured as mNIS+7 composite score was halted rather than slowed following the switch to eplontersen. Similarly, in contrast to historical placebo which showed progressive decline, patient QoL (Norfolk QoL-DN total score) appeared stabilized and maintained during the eplontersen treatment period, consistent with eplontersen treatment in NEURO-TTRansform and inotersen treatment in NEURO-TTR ﻿[19, 20]. mBMI was used to evaluate nutritional status to avoid potential discrepancies that can occur with BMI measurement due to a low serum albumin level and fluid retention [23]. In this analysis, the evident decline in nutritional status with inotersen was similar to that seen for historical placebo while the dramatic return of nutritional status to baseline following switch to eplontersen was consistent with that seen in the NEURO-TTRansform trial, where mBMI was maintained at baseline levels during 85 weeks of eplontersen treatment [19].

The general improvement in safety and tolerability profile following the switch to eplontersen treatment was likely associated with more efficient delivery to target tissues despite a lower drug dose (25-fold), lower exposure, and need for less frequent administration, while still providing comparative efficacy both to inotersen and GalNAc-conjugated ASOs as a class [24]. Specifically, in the present analysis, post-dose plasma C_trough_ levels with eplontersen were 50‒100-fold lower relative to those seen for inotersen, while virtually superimposable plasma eplontersen concentration-time profiles in the first 6 h after dose administration on Days 1, 225, and 449, suggested no accumulation of eplontersen following repeated dosing. Moreover, the pharmacokinetic profile of eplontersen was unaffected by ADAs. As the ASO-moiety of eplontersen has a virtually identical sequence and similar chemistry to inotersen, inevitably, cross-talk was evident between the inotersen and eplontersen ADA assays. Inotersen ADAs could be detected by the eplontersen ADA assay, while eplontersen ADAs against the ASO-moiety of eplontersen could be detected by the inotersen ADA assay; here, 11/13 patients with inotersen ADAs also had eplontersen ADAs while, similarly, 11/13 patients with eplontersen ADAs also had inotersen ADAs.

It is also possible that, relative to eplontersen, the comparatively poorer tolerability of inotersen might relate to high non-specific interaction/binding with proteins and potential generation of pro-inflammatory plasma cytokines; providing a milieu of immunologic dysregulation–promoting AEs such as thrombocytopenia [25]. There were very notable but opposing effects on platelet count before and following switch to eplontersen. The marked reduction in count seen during the inotersen treatment period was consistent with the NEURO-TTR trial, and the recognized risk of reduced platelet count (thrombocytopenia) with inotersen treatment [13]. In contrast, there was a gradual return to baseline level during the eplontersen treatment period, likely associated with decay of the inotersen-associated inhibitory process and the production of new platelets [20]. Similarly, this was consistent with minimal change from baseline seen for the eplontersen treatment group in the NEURO-TTRansform trial. The nature of the inhibitory process associated with ASOs that results in mild thrombocytopenia is unclear, but likely relates to innate immune activation leading to platelet sequestration [25], which differs from the mechanism underlying severe thrombocytopenia where production of antiplatelet antibodies can occur in patients with immune dysregulation [25]. Although no cases of glomerulonephritis were reported in patients randomized to inotersen, or after switching to eplontersen, a modest reduction in eGFR from baseline was seen during the inotersen treatment period (nadir ~ 5–10 mL/min/1.73 m^2^), which appeared to stabilize with no further change apparent during the eplontersen treatment period. Collectively, these observations suggest that treatment with eplontersen is not associated with any notable adverse effect on platelet count or eGFR, suggesting the possibility of foregoing the need to monitor platelet count, with a potentially significant benefit to long-term adherence to treatment.

The small study population and consequent lack of formal statistical testing might be considered limitations of this analysis. However, good similarity between observations in patients randomized to inotersen in the NEURO-TTRansform trial and to inotersen in the NEURO-TTR trial, and also between the eplontersen post-switch treatment group and patients initially randomized to eplontersen in the NEURO-TTRansform trial, provides confidence that the findings of this analysis are robust.

In summary, this prespecified analysis supports a positive benefit-risk profile for patients with ATTRv-PN who were switched from inotersen treatment to eplontersen treatment; specifically, further reduction in serum TTR concentration, benefit toward halting of neuropathy impairment and stabilization of QoL, greater nutritional status, and a more tolerable safety profile (with confirmation through more extended follow up) including restoration of platelet count to baseline.

## Supplementary Information

Below is the link to the electronic supplementary material.

Supplementary file1 (PDF 350 KB)

## Data Availability

Aggregated data may be shared with researchers following formal requests of methodologically sound proposals, under data use agreements and to the extent permissible by the informed consent documents.
