# Correction: Switching from inotersen to eplontersen in patients with hereditary transthyretin-mediated amyloidosis with polyneuropathy: analysis from NEURO-TTRansform

**DOI:** 10.1007/s00415-024-12854-8

**Published:** 2025-02-03

**Authors:** Isabel Conceição, John L. Berk, Markus Weiler, Pedro A. Kowacs, Noel R. Dasgupta, Sami Khella, Chi-Chao Chao, Shahram Attarian, T. Jesse Kwoh, Shiangtung W. Jung, Jersey Chen, Nicholas J. Viney, Rosie Z. Yu, Morie Gertz, Ahmad Masri, Márcia Waddington Cruz, Teresa Coelho

**Affiliations:** 1 ULS Santa Maria, CAML, Instituto de Medicina Molecular, Faculdade de Medicina da Universidade de Lisboa, Lisbon, Portugal; 2 Boston University School of Medicine, Boston, MA USA; 3 Amyloidosis Center and Department of Neurology, Heidelberg University Hospital, Heidelberg, Germany; 4 Instituto de Neurologia de Curitiba, Curitiba, Paraná Brazil; 5 Indiana University School of Medicine, Indianapolis, IN USA; 6 University of Pennsylvania School of Medicine, Philadelphia, PA USA; 7 National Taiwan University Hospital, Taipei, Taiwan; 8 Neuromuscular Disorders and ALS Department, Centre Hospitalier Universitaire La Timone, Marseille, France; 9 Clinical Development, Ionis Pharmaceuticals, Inc., Carlsbad, CA USA; 10 Late-Stage Development, Cardiovascular, Renal, and Metabolism, BioPharmaceuticals R&D, AstraZeneca, Gaithersburg, MA USA; 11 Preclinical Development, Ionis Pharmaceuticals, Inc, Carlsbad, CA USA; 12 Mayo Clinic, Rochester, MN USA; 13 OHSU Center for Hypertrophic Cardiomyopathy and Amyloidosis, Portland, OR USA; 14 CEPARM, Amyloidosis Center, University Hospital, Federal University of Rio de Janeiro, Rio de Janeiro, Brazil; 15 Centro Hospitalar Universitário de Santo António, Porto, Portugal

**Correction: Journal of Neurology (2024) 271:6655–6666**

 10.1007/s00415-024-12616-6

In the original version of this article, a couple of small data errors in Table 1 (corrections include adding ‘multiple’ to the ‘Other’ row, correcting values in the mNIS + 7 row and correcting one value in the Coutinho stage 2 row).

Table 1, which previously read

**Table 1:** Baseline demographics and disease characteristics

	Randomized to inotersen (*n* = 24)	Randomized to inotersen and switched to eplontersen (*n* = 20)	Randomized to eplontersen (*n* = 144)	Historical placebo (*n* = 60)
Demographics				
Mean age, years (SD)	51.1 (14.4)	51.4 (14.8)	53.0 (15.0)	59.5 (14.1)
Age group, *n* (%)				
< 65 years	16 (66.7)	13 (65.0)	100 (69.4)	34 (56.7)
65–74 years	7 (29.2)	6 (30.0)	36 (25.0)	17 (28.3)
Male, *n* (%)	16 (66.7)	13 (65.0)	100 (69.4)	41 (68.3)
Race, *n* (%)				
White	19 (82.6)	16 (80.0)	112 (78.3)	53 (88.3)
Asian	2 (8.7)	2 (10.0)	22 (15.4)	3 (5.0)
Black or African American	0	0	5 (3.5)	1 (1.7)
Other	2 (8.7)	2 (10.0)	3 (2.1)	4 (6.7)
Region, *n* (%)				
Europe	10 (41.7)	7 (35.0)	54 (37.5)	23 (38.3)
North America	5 (20.8)	5 (25.0)	21 (14.6)	26 (43.3)
South America/Australasia/Asia	9 (37.5)	8 (40.0)	69 (47.9)	11 (18.3)
Clinical characteristics				
Duration of disease from diagnosis of ATTRv-PN, years, median (range)	2.4 (0.2, 19.3)	2.2 (0.2, 19.3)	2.5 (0.0, 31.6)	2.0 (0.1, 13.3)
Duration of disease from onset of symptoms of ATTRv-PN, years, median (range)	3.5 (0.7, 47.3)	3.3 (0.7, 47.3)	4.5 (0.4, 29.5)	4.0 (0.7, 23.1)
Coutinho stage, *n* (%)				
1 (ambulatory)	18 (75.0)	15 (75.0)	115 (79.9)	42 (70.0)
2 (requires ambulatory support)	6 (25.0)	5 (25.0)	29 (20.0)	18 (30.0)
Positive ATTRv-CM diagnosis	7 (29.2)		39 (27.1)	22 (36.7)
mNIS + 7 composite score, mean (SE)	27.5 (3.1)	26.7 (3.6)	35.0 (1.7)	31.0 (2.3)
Norfolk QoL-DN total score, mean (SE)	40.1 (4.4)	37.3 (4.9)	44.1 (2.3)	48.7 (3.5)
BMI, kg/m^2^, mean (SD)	26.4 (5.4)	26.2 (4.7)	24.4 (4.9)	24.2 (4.9)
mBMI^b^ kg/m^2^ × albumin g/L, mean (SD)	1101.7 (246.5)	1098.8 (236.7)	1025.8 (235.1)	1049.9 (228.4)
V30M *TTR* variant, *n* (%)	16 (66.7)	12 (60.0)	85 (59.0)	33 (55.0)
Previous treatment with tafamidis or diflunisal	15 (62.5)	11 (55.0)	100 (69.4)	36 (60.0)

*ATTRv-CM* ATTRv cardiomyopathy, *ATTRv-PN* ATTRv polyneuropathy, *BMI* body mass index, *mBMI* modified BMI, *mNIS + 7* modified Neuropathy Impairment Score + 7, *Norfolk QoL-DN* Norfolk Quality of Life-Diabetic Neuropathy, *SD* standard deviation, *SE* standard error, *TTR* transthyretin

^a^From the NEURO-TTRansform study [19]

^b^Defined as BMI (kg/m^2^) × albumin level (g/L); higher scores are indicative of better nutritional status

Should have read

**Table 1:** Baseline demographics and disease characteristics

	Randomized to inotersen (*n* = 24)	Randomized to inotersen and switched to eplontersen (*n* = 20)	Randomized to eplontersen (*n* = 144)^a^	Historical placebo (*n* = 60)^a^
Demographics				
Mean age, years (SD)	51.1 (14.4)	51.4 (14.8)	53.0 (15.0)	59.5 (14.1)
Age group, *n* (%)	24 (100)	20 (100)	144 (100)	60 (100)
< 65 years	16 (66.7)	13 (65.0)	100 (69.4)	34 (56.7)
65–74 years	7 (29.2)	6 (30.0)	36 (25.0)	17 (28.3)
Male, *n* (%)	16 (66.7)	13 (65.0)	100 (69.4)	41 (68.3)
Race, *n* (%)	23 (95.8)	20 (100)	143 (99.3)	60 (100)
Asian	2 (8.7)	2 (10.0)	22 (15.4)	3 (5.0)
Black or African American	0	0	5 (3.5)	1 (1.7)
White	19 (82.6)	16 (80.0)	112 (78.3)	53 (88.3)
Other or multiple	2 (8.7)	2 (10.0)	4 (2.8)	3 (5.0)
Region, *n* (%)	24 (100)	20 (100)	144 (100)	60 (100)
Europe	10 (41.7)	7 (35.0)	54 (37.5)	23 (38.3)
North America	5 (20.8)	5 (25.0)	21 (14.6)	26 (43.3)
South America/Australasia/Asia	9 (37.5)	8 (40.0)	69 (47.9)	11 (18.3)
Clinical characteristics				
Duration of disease from diagnosis of ATTRv-PN, years, median (range)	2.4 (0.2, 19.3)	2.2 (0.2, 19.3)	2.5 (0.0, 31.6)	2.0 (0.1, 13.3)
Duration of disease from onset of symptoms of ATTRv-PN, years, median (range)	3.5 (0.7, 47.3)	3.3 (0.7, 47.3)	4.5 (0.4, 29.5)	4.0 (0.7, 23.1)
Coutinho stage, *n* (%)	24 (100)	20 (100)	144 (100)	60 (100)
1 (ambulatory)	18 (75.0)	15 (75.0)	115 (79.9)	42 (70.0)
2 (requires ambulatory support)	6 (25.0)	5 (25.0)	29 (20.1)	18 (30.0)
Positive ATTRv-CM diagnosis	7 (29.2)		39 (27.1)	22 (36.7)
mNIS + 7 composite score, mean (SE)	65.1 (6.8)	65.6 (8.2)	81.3 (3.6)	74.8 (5.0)
Norfolk QoL-DN total score, mean (SE)	40.1 (4.4)	37.3 (4.9)	44.1 (2.3)	48.7 (3.5)
BMI, kg/m^2^, mean (SD)	26.4 (5.4)	26.2 (4.7)	24.4 (4.9)	24.2 (4.9)
mBMI^a^ kg/m^2^ × albumin g/L, mean (SD)	1101.7 (246.5)	1098.8 (236.7)	1025.8 (235.1)	1049.9 (228.4)
V30M *TTR* variant, *n* (%)	16 (66.7)	12 (60.0)	85 (59.0)	33 (55.0)
Previous treatment with tafamidis or diflunisal	15 (62.5)	11 (55.0)	100 (69.4)	36 (60.0)

*ATTRv-CM* ATTRv cardiomyopathy, *ATTRv-PN* ATTRv polyneuropathy, *BMI* body mass index, *mBMI* modified BMI, *mNIS + 7* modified Neuropathy Impairment Score + 7, *Norfolk QoL-DN* Norfolk Quality of Life-Diabetic Neuropathy, *SD* standard deviation, *SE* standard error, *TTR* transthyretin

^a^Defined as BMI (kg/m^2^) × albumin level (g/L); higher scores are indicative of better nutritional status

The original article has been corrected.

