# Supplementary material for: Switching from inotersen to eplontersen in patients with hereditary transthyretin-mediated amyloidosis with polyneuropathy: analysis from NEURO-TTRansform
Source: J Neurol. 2024 Aug 13;271(10):6655–66. doi: 10.1007/s00415-024-12616-6 (PMC11447117; doi:10.1007/s00415-024-12616-6)
Supplement: Supplementary file 1 — Supplementary file1 (PDF 350 KB) [file 415_2024_12616_MOESM1_ESM.pdf]

Switching from inotersen to eplontersen in patients with hereditary transthyretin-mediated amyloidosis with polyneuropathy: Analysis from NEURO-TTRansform

Journal: *Journal of Neurology*

Authors: Isabel Conceição, John L. Berk, Markus Weiler, Pedro A. Kowacs, Noel R. Dasgupta, Sami Khella, Chi-Chao Chao, Shahram Attarian, T. Jesse Kwoh, Shiangtung W. Jung, Jersey Chen, Nicholas J. Viney, Rosie Z. Yu, Morie Gertz, Ahmad Masri, Márcia Waddington Cruz, Teresa Coelho

Corresponding author: Isabel Conceição

ULS Santa Maria, CAML, Instituto de Medicina Molecular, Faculdade de Medicina da Universidade de Lisboa, Portugal.

Tel: +351 21 798 5100; E-mail: imsconceicao@gmail.com

Supplementary materials

Supplementary Fig. 1. NEURO-TTRansform study design

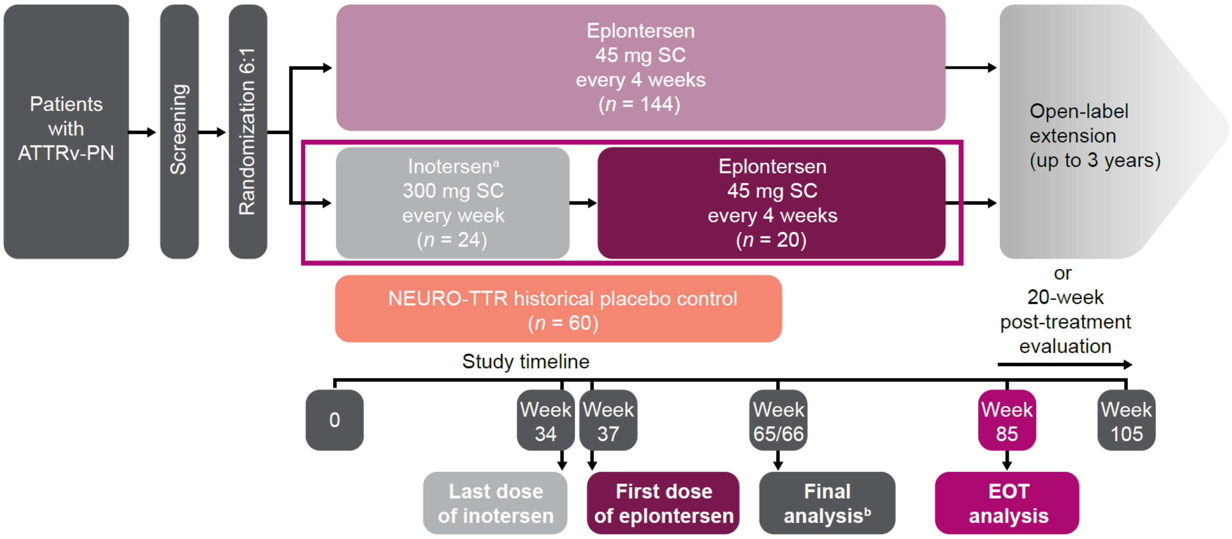

[a]Inotersen reference group was intended to confirm sufficiently comparable disease progression and treatment response patterns between NEURO-TTR (NCT01737398) and NEURO-TTRansform.

[b]Final analysis refers to final historical placebo-controlled efficacy analysis.

ATTRv-PN, hereditary transthyretin amyloidosis with polyneuropathy; EOT, end-of-treatment; SC, subcutaneous.

Supplementary Fig. 2. eGFR in patients randomized to inotersen who then switched to eplontersen

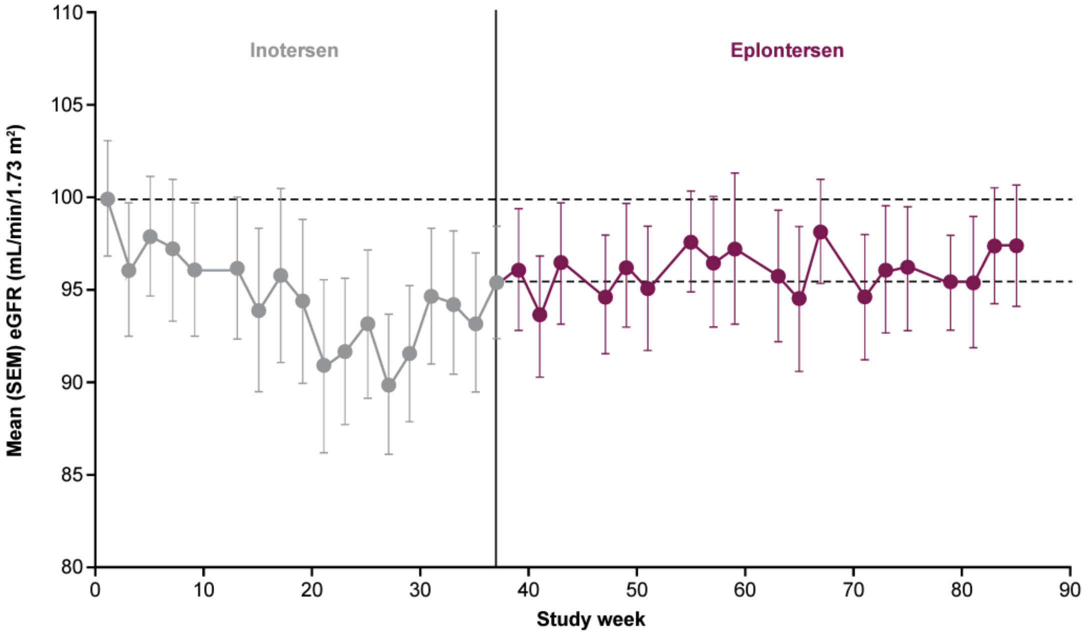

eGFR, estimated glomerular filtration rate; SEM, standard error of the mean.
